# Identification of a Novel Ferroptosis-Related Gene Prediction Model for Clinical Prognosis and Immunotherapy of Colorectal Cancer

**DOI:** 10.1155/2021/4846683

**Published:** 2021-11-24

**Authors:** Ya-bing Yang, Jia-xin Zhou, Sheng-hui Qiu, Jia-shuai He, Jing-hua Pan, Yun-long Pan

**Affiliations:** ^1^Department of General Surgery, The First Affiliated Hospital of Jinan University, Guangzhou 510632, China; ^2^International School, Jinan University, Guangzhou, Guangdong 510632, China; ^3^MOE Key Laboratory of Tumor Molecular Biology and Key Laboratory of Functional Protein Research of Guangdong Higher Education Institutes, Institute of Life and Health Engineering, Jinan University, Guangzhou 510632, China

## Abstract

**Background:**

Colorectal cancer (CRC) is the third most common malignancies worldwide. Ferroptosis is a programmed, iron-dependent cell death observed in cancer cells. However, the prognostic potential and immunotherapy biomarker potential of ferroptosis-related genes (FRGs) in CRC patients remains to be clarified.

**Methods:**

At first, we comprehensively analysed the different expression and prognosis of related FRGs in CRC patients based on TCGA cohort. The relationship between functional enrichment of these genes and immune microenvironment in CRC was investigated using the TCGA database. Prognostic model was constructed to determine the association between FRGs and the prognosis of CRC. Relative verification was done based on the GEO database. Meanwhile, the ceRNA network of FRGs in the model was also performed to explore the regulatory mechanisms.

**Results:**

Eight differentially expressed FRGs were associated with the prognosis of CRC patients. Patients from the TCGA database were classified into the A, B, and C FRG clusters with different features. And FRG scores were constructed to quantify the FRG pattern of individual patients with colorectal cancer. The CRC patients with higher FRG score showed worse survival outcomes, higher immune dysfunction, and lower response to immunotherapy. The prognostic model showed a high accuracy for predicting the OS of CRC. Finally, a ceRNA network was analysed to show the concrete regulation mechanisms of critical FRGs in CRC.

**Conclusions:**

The FRG risk score prognostic model based on 8 FRGs exhibit superior predictive performance, providing a novel prognostic model with a high accuracy for CRC patients. Moreover, FRG score can be the potential biomarker of the response of immunotherapy for CRC.

## 1. Introduction

Colorectal cancer (CRC) is the third most common malignancy worldwide and has become one of the leading causes of cancer-related death [1, 2]. In the past few years, the incidence of CRC has kept increasing in Asian countries, including China, Singapore, South Korea, and Japan, owing to demographic trends and adaptation to western lifestyles [3]. Traditional therapies, such as surgical resection, chemotherapy, radiotherapy, and combined therapy, are currently used for the treatment of CRC. However, the therapeutic efficiency of these therapies is severely limited due to the complexity of CRC pathogenesis and treatment resistance [4]. Therefore, there exists an urgent need to detect molecular changes and explore molecular mechanisms which are involved in tumorigenesis and are related with the prognosis of CRC. What is more, nowadays, immunotherapy has become a novel treatment for many cancers. There grows a high enthusiasm in immunotherapy research, with loads of treatments in clinical and preclinical developing. It becomes a powerful therapy to improve the prognosis of some solid malignant tumors [5]. Various experiments and clinical studies have demonstrated that immunotherapy does have effective advantages over traditional antitumor therapy, which can helpfully improve cancer prognosis condition. The identification of novel biomarkers may provide new immunotherapy markers for CRC treatment, which may assist the cure of CRC. For example, some researches have already found that immune biomarkers include programmed cell death-1 (PD-1), infiltration of the CD8+ T-cell, PD ligand 1 (PD-L1) expression, and tumor mutation burden (TMB) for immunotherapy in CRC. However, there also exists some problems. Take PD-L1biomarker as an example, the heterogeneity of PD-L1 detection is an important issue at present. Different antibodies for detecting PD-L1 expression, different detection platforms, and different evaluation systems may have different positive critical values, making it difficult to form a consistent standard to measure pD-L1 expression in tumor cells. So, it is necessary for human to learn more about biomarkers and CRC. Meanwhile, the prognosis of colorectal cancer can be improved a lot due to an earlier diagnosis through biomarkers [6], which also certifies the significance and necessity of our research.

Ferroptosis is a programmed, iron-dependent cell death driven by the accumulation of lipid peroxides. It differs from autophagy, apoptosis, and other regulated cell death [7, 8]. The morphology of mitochondria undergoes dramatic changes during ferroptosis, including the loss of mitochondria crista, mitochondrial shrinkage with increased membrane density, and outer mitochondrial membrane rapture [9]. In recent years, ferroptosis has emerged as a promising treatment concern for cancer therapy, especially in cancers resistant to conventional therapies [10, 11]. Meanwhile, several studies have suggested the use of ferroptosis-related gene (FRG) signatures as a prognostic feature for hepatocellular carcinoma [12, 13]. Previous evidence also suggested that ferroptosis plays an important role in CRC. For example, RSL3 triggers ferroptosis by upregulating LIP and promoting the accumulation of ROS in cells [14]. The level of ACADSB in CRC tissues is lower than that of normal colon tissues and is correlated with lower TNM stage in CRC patients [15]. However, the characterization of FRGs in CRC tumorigenesis and their prognostic potential for CRC warrant further investigation.

By using bioinformatics techniques, researchers can use public database data to identify genes signature associated with prognosis in colorectal cancer, and noncoding RNAs such as lncRNAs are also closely related to tumor characteristics [16]. In this study, we identified eight meaningful ferroptosis-related gene signatures from public databases and constructed a prognostic model for CRC with high accuracy. Moreover, to discuss about the CRC immunotherapy, we estimated the TIDE value and immunotherapy sensitivity of FRGs, which may provide potential biomarkers for clinical treatment and prognosis. To further study the mechanism of CRC, we created the circRNA-miRNA-lncRNA-mRNA network regulated FRGs in the prognostic model.

## 2. Materials and Methods

### 2.1. Data Acquisition

The RNA sequencing and corresponding clinical data were downloaded from the TCGA database (https://portal.gdc.cancer; including 568 CRC samples and 44 normal tissue samples) and the GEO database (http://www.ncbi.nlm.nih.gov/geo/; GSE17536, including 177 CRC samples). And microarray datasets which provide circRNA expression data in CRC patients were acquired from the GEO database (GSE17536, including 10 CRC samples and 10 corresponding normal tissue samples). A total of 60 FRGs have been retrieved from previous studies [7, 10, 17, 18]. Since both the TCGA database and the GEO database are publicly available and this study strictly followed access policies for databases and publication guidelines, ethical approval from a local ethics committee was not required.

### 2.2. Screening and Identifying DEGs Associated with CRC Prognosis

The mRNA sequencing data from the TCGA database was matched with FRGs. The differentially expressed genes (DEGs) between CRC tissues and adjacent nontumorous tissues were identified by the “limma” R package with a false discovery rate of <0.05. Univariate Cox analysis of overall survival (OS) was performed using the “survival” R package to screen FRGs with prognostic potential.

### 2.3. Consensus Clustering Analysis and Construction of FRG Score

In order to investigate the function of FRGs in CRC, the prognostic DEGs were incorporated to divide tumor samples into different clusters with “ConsensusClusterPlus” R package. Kaplan–Meier analysis was used to evaluate the differences of OS between different groups. Thereafter, PCA (principal component analysis) was used to validate the reliability of clustering with the R package “ggplot2.” To investigate the difference on biological process between different groups, we performed GSVA enrichment analysis through “GSVA” R packages.

Then, we constructed a set of scoring system to evaluate the FRGs pattern of individual CRC patient based on principal component analysis. This score is termed as FRG score. Both principal component 1 and 2 were selected to act as scores. The infiltration of immune cells was assessed between patients with different FRG scores with CIBERSORT computational method.

### 2.4. Immunotherapy Response Predictions

TIDE (http://tide.dfci.harvard.edu/) is a computational method that integrates the expression signatures of T cell dysfunction and exclusion to model tumor immune evasion. We used the TIDE algorithm to predict the clinical response to immune checkpoint blockade (ICB) in CRC patients based on pretreatment genomics.

### 2.5. Correlation between FRG Score and Tumor Mutational Burden (TMB)

We analysed the distribution differences of somatic mutation using maftools package in the TCGA cohort. A correlation analysis was also performed to further reveal the concrete association between FRG score and tumor mutation.

### 2.6. Development of Prognostic Signatures Based on FRGs

The FRGs were incorporated into the LASSO Cox regression using the “glmnet” R package. In order to prevent overfitting effects of the model, the penalty regularization parameter *λ* was determined via the ten-fold cross validation. The risk score of the FRG model for each patient was calculated as follows: RiskScore = ∑_*i*=1_^*n*^(Expi∗*βi*). Where *n* is the number of selected FRGs, Expi is the expression value of gene *i*, and *βi* is the coefficient of gene *i* generated from LASSO regression analysis. To determine whether the risk score was an independent prognostic predictor for OS, compared to other clinical features, univariate and multivariate Cox regression analyses were performed.

### 2.7. Construction and Evaluation of the Nomogram

The “rms” R package was used to construct a predictive nomogram and corresponding calibration maps based on independent predictive factors. Time-dependent receiver operating characteristic (ROC) curve analysis was performed to evaluate the predictive power of the nomogram using the “timeROC” R package. Patients from GSE39582 were analysed using the same formula as that for the TCGA database. ROC curves were generated to determine the sensitivity and specificity of the predictive nomogram.

### 2.8. Construction of circRNA–miRNA–lncRNA–mRNA Network

Differential lncRNAs and miRNAs were screened between tumorous and nontumorous samples of CRC patients from TCGA cohorts, and miRNAs targeting FRGs in prognostic models and lncRNAs were identified by GDCRNATools. Tumor and nontumor samples of GSE126094 were used to screen for circRNA with abnormal expression in tumors. The circRNA bound to miRNA was predicted using Starbase database (http://starbase.sysu.edu.cn/). Finally, the intersection of circRNA–miRNA, miRNA-lncRNA, and miRNA–mRNA pair was taken to construct the circRNA–miRNA–lncRNA–mRNA regulatory network.

### 2.9. Statistical Analysis

All statistical analyses were performed using the R software (Version 4.0.3). The Student's two-sided *t*-test was performed to compare gene expression between CRC tissues and adjacent nontumorous tissues. The OS of different groups was compared by Kaplan-Meier analysis followed by log-rank test. All *P* values were two-tailed. *P* value < 0.05 was considered statistically significant if not specified above.

## 3. Results

### 3.1. Identification of Ferroptosis-Related Prognostic DEGs in the TCGA Database

**Figure 1:**
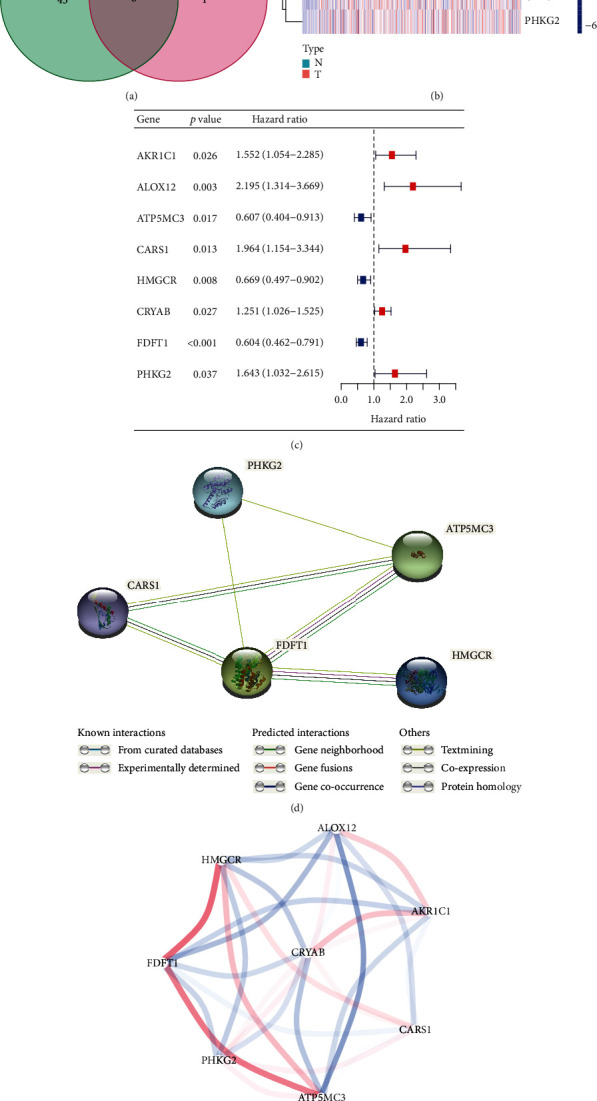
Identification of the candidate ferroptosis-related genes in the TCGA database. (a) Venn diagram to identify differentially expressed genes between tumor and adjacent normal tissue that were correlated with OS. (b) The 8 overlapping genes express quantity in tumor tissue. (c) Forest plots showing the results of the univariate Cox regression analysis between gene expression and OS. (d) The PPI network downloaded from the STRING database indicated the interactions among the candidate genes. (e) The correlation network of candidate genes. The correlation coefficients are represented by different colors.

There were 51 out of 60 FRGs differentially expressed between CRC tissues and adjacent nontumorous tissues, and 9 of them were associated with the OS of CRC patients (Figure 1(a)). Thus, 8 of the DEGs (AKR1C1, ALOX12, ATP5MC3, CARS1, HMGCR, CRYAB, FDFT1, and PHKG2) related with prognosis of CRC patients were chosen (Figures 1(b) and 1(c)). The interaction network of these genes (Figure 1(d)) indicated that CARS1, ATP5MC3, and FDFT1 are the pivot genes. The correlations among these genes are shown in Figure 1(e).

### 3.2. FRG Clusters Mediated by Prognostic Differentially Expressed Genes

**Figure 2:**
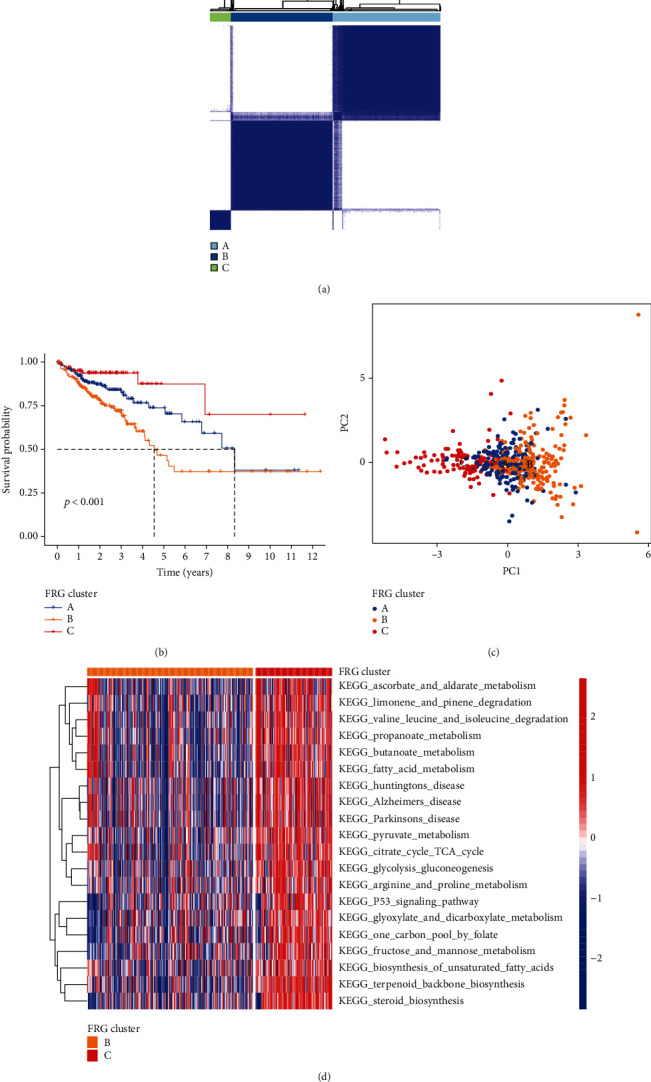
Clusters of FRG expression in CRC patients and biological characteristics. (a) Three distinct FRG clusters were identified using unsupervised clustering. (b) Survival analyses for the three FRG clusters based on patients with colorectum cancer. (c) Principal component analysis for the transcriptome profiles of three FRG clusters. (d) GSVA enrichment analysis showing the activation states of biological pathways in cluster B vs. cluster C. The heat map was used to visualize these biological processes, red represented activated pathways, and blue represented inhibited pathways.

The R package of ConsensusClusterPlus was used to classify patients with qualitatively different ferroptosis patterns based on the expression of 8 prognostic differentially expressed FRGs, and three distinct FRG patterns were eventually identified using unsupervised clustering. We termed these patterns as FRG cluster A-C, respectively (Figure 2(a)). Prognostic analysis for the three FRG clusters revealed the particularly prominent survival advantage in cluster C, as the contrast, cluster B had the worst prognosis (*P* < 0.001) (Figure 2(b)). Principal component analysis for the transcriptome profiles of the three FRG patterns is showing a remarkable difference on transcriptome between cluster B and C (Figure 2(c)). To explore the biological process among cluster B and C, we performed gene set variation analysis (GSVA). As shown in Figure 2(d), cluster C was markedly enriched in metabolism, especially in lipid metabolism. Since ferroptosis was associated with lipid peroxides, differences in lipid metabolism among patients with different prognostic suggest differences ferroptosis status.

### 3.3. Correlation between FRG Score and Prognosis in CRC Patients

**Figure 3:**
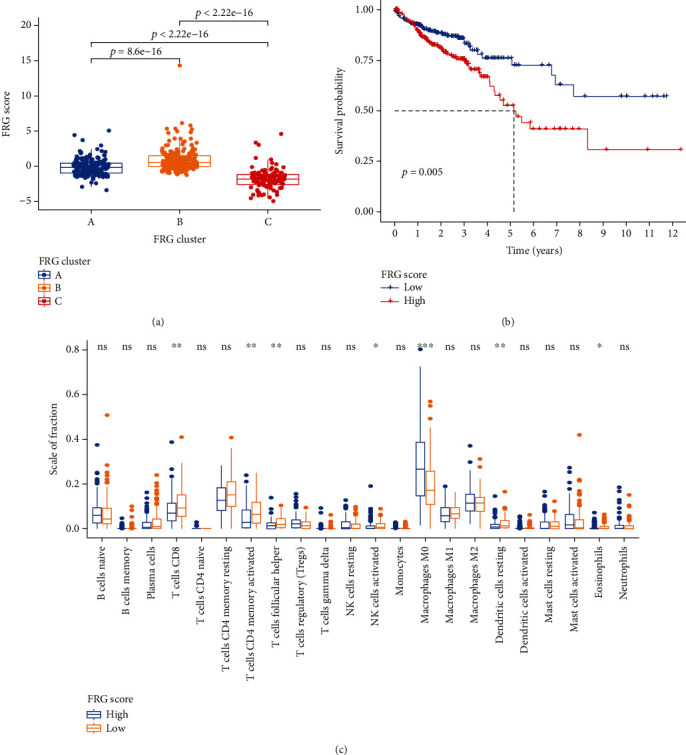
Construction FRG score. (a) Differences in FRG score among three FRG Clusters. (b) Kaplan-Meier curves indicated FRG score was markedly related to overall survival of patients in the TCGA cohort. (c) Different immune cell subset infiltration of low and high FRG score patient groups.

Considering the individual heterogeneity of the expression of FRGs, we constructed a set of scoring system to quantify the FRG pattern of individual patients with colorectal cancer based on the 8 prognostic differentially expressed FRGs. We termed it as FRG score. Patients of three clusters were divided in two groups according to the FRG scores. Meanwhile, their prognosis and biological process of CRC were further evaluated based on the score. The results showed that these CRC patients with higher FRG scores showed a worse survival outcome than those patients with lower FRG scores (*P* = 0.005) (Figures 3(a) and 3(b)).

Moreover, to investigate the association between FRG score and immune status, the CIBERSORT computational method was used to quantify different immune cell subsets and cell functions. The results revealed that the grade of CD8+ T cells in higher FRG score group was lower than the lower FRG score group (Figure 3(c)), which indicates the FRG score is related to the immune microenvironment of CRC.

### 3.4. FRG Score Is a Biomarker for Immune Checkpoint Therapy in CRC Patients

**Figure 4:**
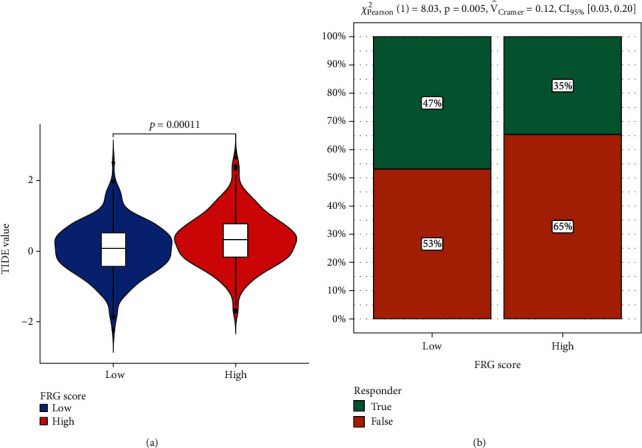
Predictions of the immunotherapy response in CRC patients. (a) The violin plots present of immune dysfunction in high and low FRG score groups. (b) The likelihood of the clinical response to antiPD1 and anti-CTLA4 therapy for high and low FRG score patients from the TCGA cohorts. True represents immunotherapy responders, while false represents immunotherapy nonresponders.

Afterward, the tumor immune dysfunction and exclusion (TIDE) algorithm was used to predict the immune checkpoint therapy response based on FRG score in CRC patients. Interestingly, according to the results shown in Figures 4(a) and 4(b), CRC patients with lower FRG score had less immune dysfunction and were more likely to respond to immunotherapy. Suggest that FRG score can be used as reference index for clinical treatment of colorectal cancer patients, as whether use immunotherapy for these patients.

### 3.5. FRG Score Is Correlated with TMB in CRC Patients

**Figure 5:**
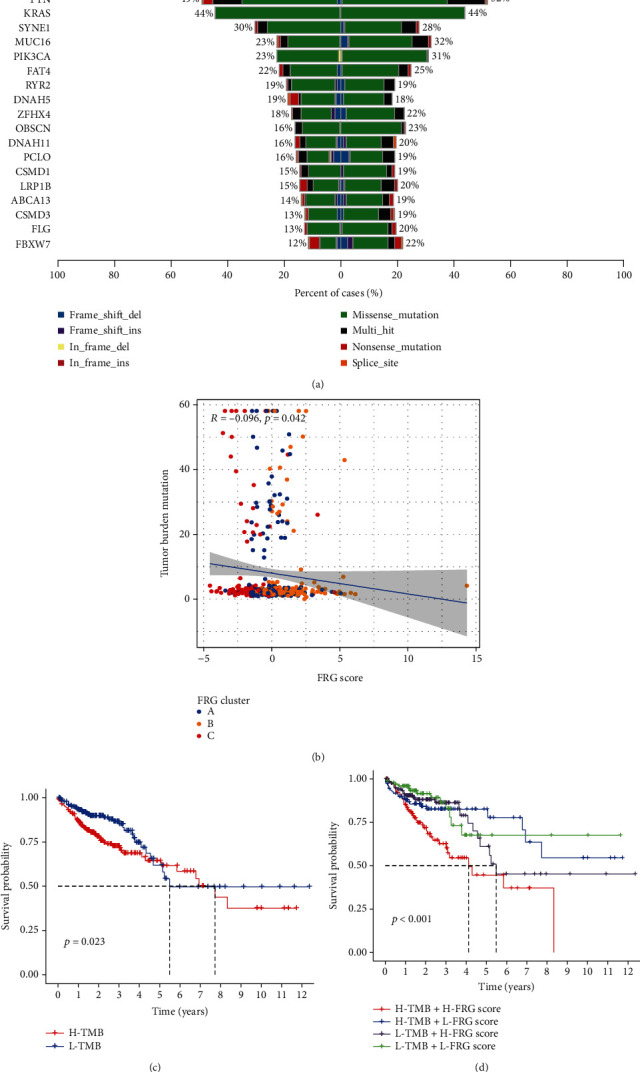
Correlation of FRG score with TMB. (a) Tumor somatic mutation established by those with high and low FRG score. (b) Correlations between FRG score and TMB. (c) Survival analyses for low and high TMB patient groups in the TCGA cohort using Kaplan-Meier curves. (d) Survival analyses for subgroup patients stratified by both FRG score and TMB using Kaplan-Meier curves.

As the TMB is a critical biomarker for immune checkpoint therapy of CRC patient, thus, we analysed the distribution differences of somatic mutation between low and high FRG score in TCGA cohort of CRC using maftools package. As shown in Figure 5(a), low FRG score group presented more extensive tumor mutation burden than the high FRG score group. The FRG score and TMB also exhibited a significant negative correlation in TCGA cohort (*P* = 0.042) (Figure 5(b)). CRC patients with high TMB level had worse prognosis in the TCGA cohort (Figure 5(c)). Moreover, we also combined the FRG score and TMB level for predicting the prognosis of CRC. Interestingly, we found that CRC patients with high FRG score and high TMB level had the worst prognosis (Figure 5(d)).

### 3.6. FRG Risk Score Is an Independent Biomarker for Prognosis of CRC Patients

**Figure 6:**
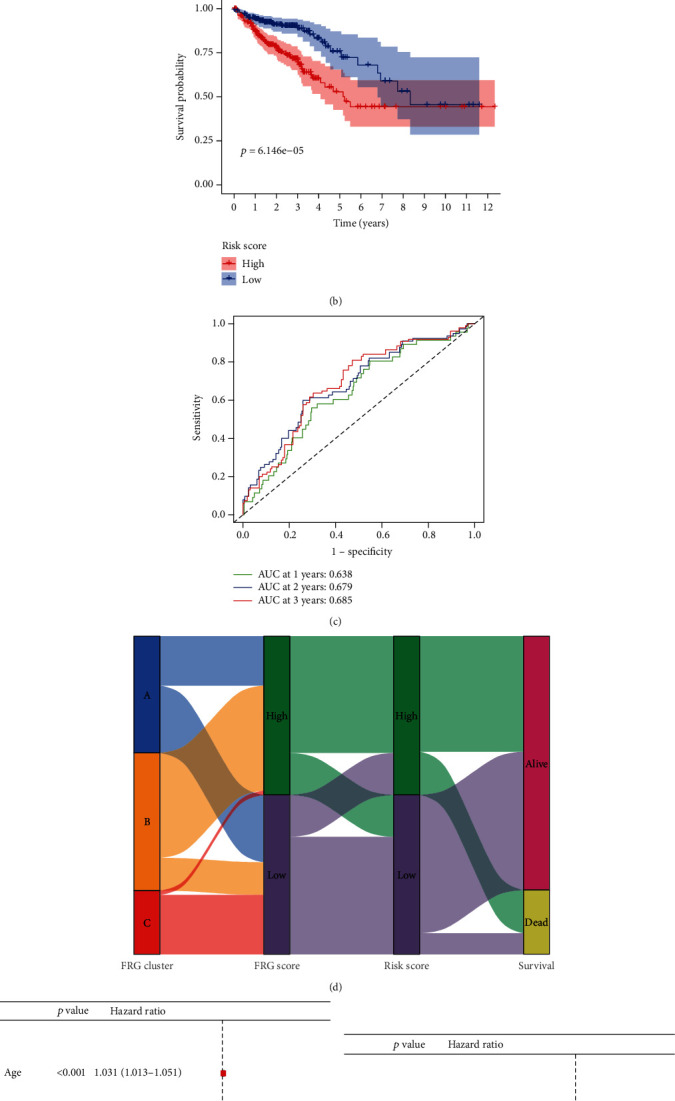
Prognostic analysis of the 8-gene signature model in the TCGA cohort. (a) The distribution and median value of the risk scores in the TCGA cohort. (b) Kaplan-Meier curves for the OS of patients in the high-risk group and low-risk group in the TCGA cohort. (c) AUC of time-dependent ROC curves verified the prognostic performance of the risk score in the TCGA cohort. (d) Alluvial diagram showing the changes of FRG clusters, FRG score, risk score, and survival state. (e) and (f) Results of the univariate (e) and multivariate (f) Cox regression analyses regarding OS in the TCGA derivation cohort.

The expression profile of the eight FRGs was used to establish the prognosis model using LASSO Cox regression analysis (Figure 6(a)). The risk score was calculated as follows: Risk Score = SUM (0.21∗AKR1C1 + 0.573∗ALOX12–0.062∗ATP5MC3 + 0.543∗CARS1–0.11∗HMGCR + 0.182∗CRYAB–0.3∗FDFT1 + 0.348∗PHKG2). The Kaplan-Meier curve revealed that the prognosis of low-risk patients was significantly better than that of the high-risk group (Figure 6(b)), suggesting the great sensitivity and specificity of the prognostic signature in predicting CRC survival outcome. The predictive performance of the risk score for OS was evaluated by time-dependent ROC curves, and the area under the curve (AUC) reached 0.638 at 1 year, 0.679 at 2 years, and 0.685 at 3 years, showing a high accuracy (Figure 6(c)). In order to study the relationship of patients in different ferroptosis assessment systems, we constructed an alluvial diagram showed the changes and associations of FRG clusters, FRG score, risk score, and survival state (Figure 6(d)). Univariate and multivariate Cox regression analyses were then performed to determine whether FRG risk score can be a predictor for OS, independent of other clinical features (including gender, age, and TNM stage). And we found that TNM stage (HR = 2.089), age (HR = 1.038), and risk score (HR = 1.858) were independent predictors for OS (Figures 6(e) and 6(f)).

### 3.7. Construction and Validation of the FRG Risk Score Nomogram

**Figure 7:**
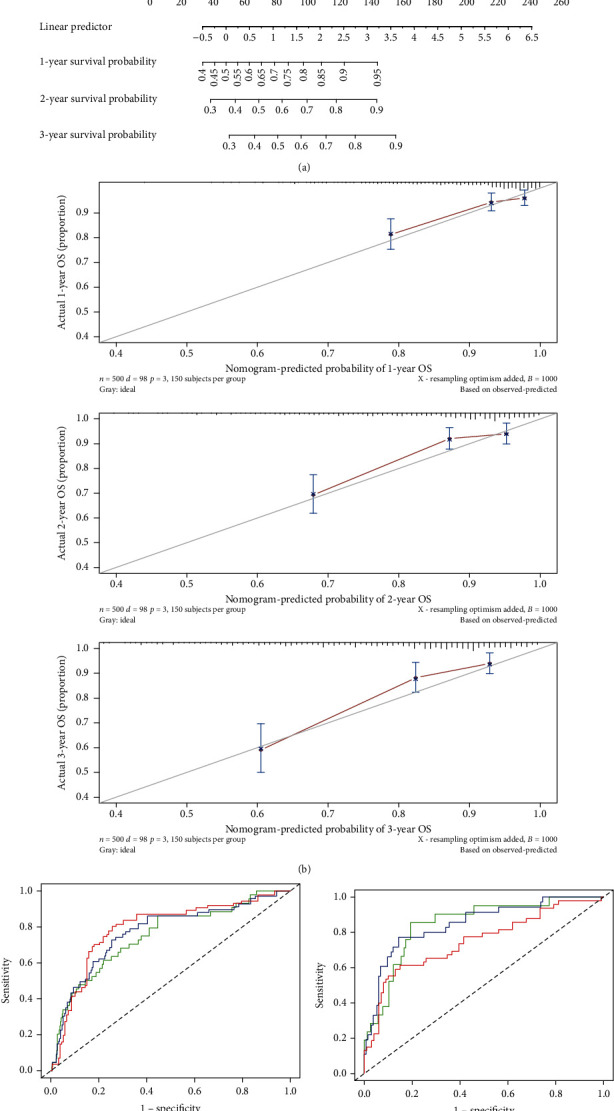
Construction and validation of a predictive nomogram. (a) The nomogram for predicting the OS of patients with GC at 1, 2, and 3 years. (b) Calibration curves of the nomogram for OS prediction at 1, 2, and 3 years. (c) ROC curves to evaluate the predictive ability of the nomogram in the TCGA cohort. (d) ROC curves to examine the robustness of the model based on GEO database.

Based on the above prognostic factors, a nomogram which also including FRG risk score was developed to quantify the prediction of individual survival probability for 1, 2, and 3 years (Figure 7(a)). The C-index of the nomogram was 0.75 (95% CI: 0.70–0.81). The calibration curves indicated great consistency between predicted OS and actual observation at 1, 2, and 3 years (Figure 7(b)).

Then, ROC curves were generated to verify the predictive value of the nomogram. The AUCs for 1-year, 2-year, and 3-year OS were 0.752, 0.771, and 0.791, respectively, in the TCGA database (Figure 7(c)). To examine the robustness of the model, we incorporated patients from the GEO database into the predictive model as a verification. The results showed that the AUCs for 1-year, 2-year, and 3-year OS with the nomogram were 0.850, 0.849, and 0.749, respectively (Figure 7(d)).

### 3.8. The ceRNA Network of the Key Genes of FRG Risk Score Model

**Figure 8:**
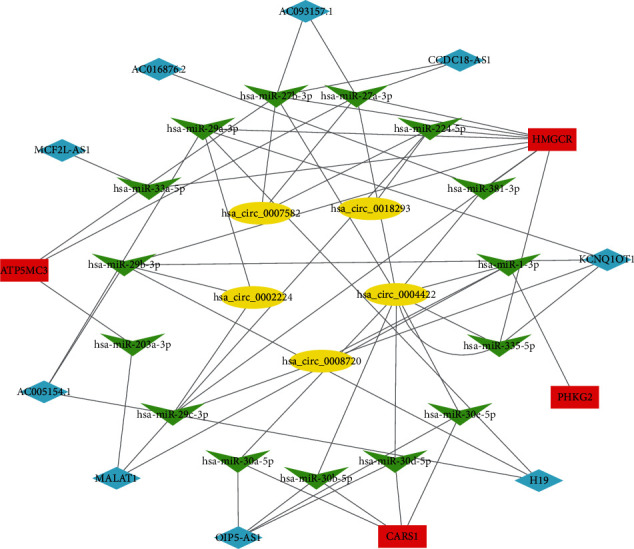
Construction of the circRNA–miRNA–lncRNA–mRNA regulatory network.

As noncoding RNAs play important roles in the regulation of expression of genes. ceRNA network was performed to learn about the concrete mechanism. We used GDCRNATools to find 15 miRNAs, which consider 4 FRGs as target in prognosis model, and 5 lncRNAs binds to the miRNAs. Then, we found 5 different expressed circRNAs which had spongy effect to the miRNAs in Starbase. In addition, we constructed the circRNA–miRNA–lncRNA–mRNA regulatory network, which showed a regulatory network of the FRG risk score model based on ceRNA network in CRC (Figure 8).

## 4. Discussion

Colorectal cancer is the third most common cancer all around the world, with 1.36 million people diagnosed in 2012 [6]. In this study, we analysed the expression of 60 FRGs in CRC tissues and investigated their association with the OS prognosis of CRC patients by estimating the data from public databases. Eight differentially expressed FRGs were finally selected, which are associated with the prognosis in CRC. Patients with different expression level of these genes showed diverse functional enrichment and immune status. In addition, we proposed a prognostic nomogram based on these genes, which exhibited a great sensitivity and specificity in predicting the overall survival of 1, 2, and 3 years with a high accuracy.

In our study, different FRG clusters showed significantly distinct overall survival, suggesting that ferroptosis status was significantly correlated with prognosis. Cluster C had the lowest FRG score and was markedly enriched in metabolism, especially in lipid metabolism. Lipid oxidation plays a central role in the process of ferroptosis, while normal lipid metabolism might be expected to perturb ferroptotic cell death [19]. Interestingly, not only did we observed better outcomes in the low FRG score group patients, but we also observed more CD8^+^ T cell infiltration in them. Effector CD8+ T cells released cytokines IFN*γ*, and both of them were key features of effective immunotherapy in cancer patients [20]. And the low FRG score group patients were more likely to respond to immunotherapy in our study, suggesting that ferroptosis may be a potential biomarker for cancer immunotherapy.

The potential modulation between ferroptosis and tumor immunity remains unclear. TMB associated with FRG score may be a potential mechanism. There were more somatic mutations in the low FRG score group, and FRG score was negatively correlated with TMB in TCGA. Somatic mutations in tumor DNA produce neoantigens, with mutation-derived antigens recognized and targeted by the immune system, especially after treatment with drugs that activate T cells. The more somatic mutations, the more neoantigens to form [21]. The mechanism between TMB and ferroptosis has not been reported. However, previous studies have been reported that radiotherapy not only increases somatic mutations but also induces tumor ferroptosis. The connection needs to be further studied [22].

Our prognostic model consisted of eight FRGs (AKR1C1, ALOX12, ATP5MC3, CARS1, HMGCR, CRYAB, FDFT1, and PHKG2), which can be roughly classified into five categories: lipid metabolism (AKR1C1, ALOX12, HMGCR, and FDFT1), energy metabolism (ATP5MC3), antioxidant metabolism (CARS1), intracellular architecture (CRYAB), and andiron metabolism (PHKG2). AKR1C1 is a member of the Aldo-keto reductase superfamily, which is implicated in the elimination of the final products of lipid peroxidation and can enhance the detoxification effect of active aldehydes generated by plasma membrane oxidation during ferroptosis [23]. ALOX12, a member of the lipoxygenase family, is upregulated in CRC tissues and promotes the production of tumor stromal vascular endothelial growth factor, resulting in angiogenesis and tumor metastasis [24, 25]. The overexpression of ALOX12 makes cells sensitive to ferroptosis [26]. ATP5MC3, also known as ATP5G3, is a subunit of mitochondrial ATP synthase. Sorafenib has been shown to alter mitochondrial morphology, decrease oxidative phosphorylation, induce the collapse of mitochondrial membrane potential, reduce ATP synthesis, and ultimately lead to ferroptosis [27]. CARS1 encodes class 1 aminoacyl-tRNA synthetase. The knockdown of CARS1 has been reported to inhibit ferroptosis induced by cystine deprivation [28]. HMGCR is a rate-limiting enzyme in cholesterol synthesis, and the inhibition of HMGCR enhances FIN-56-induced ferroptosis [29]. CRYAB is a structural protein of the crystalline lens that participates in the intracellular architecture. High CRYAB level is associated with a poor prognosis in CRC [30]. FDFT1 encodes the first specific enzyme in cholesterol biosynthesis, and the knockdown or inhibition of FDFT1 represses FIN-56-induced ferroptosis [31]. PHKG2 encodes a subunit of phosphorylase kinase, and the knockdown of PHKG2 inhibits ferroptosis induced by erastin [32]. In our study, five of these genes (AKR1C1, ALOX12, CARS1, CRYAB, and PHKG2) were associated with a poor prognosis in CRC, while the remaining three (ATP5MC3, HMGCR, and FDFT1) were associated with a better prognosis. AKR1C1, ALOX12, CARS1, and PHKG2 have been reported to enhance ferroptosis. However, whether these genes would affect the prognosis of CRC patients by regulating ferroptosis remains to be elucidated.

Our prognostic model showed a high accuracy in CRC outcomes prediction. Meanwhile, four of the FRGs, ATP5MC3, HMGCR, CARS1, and PHKG2, are strongly related to the competitive endogenous RNA (ceRNA) network, which has become more and more popular in cancer research nowadays. ceRNA can regulate the expression of mRNA through the competitive binding of the noncoding RNAs. miRNAs direct the RNA-induced silencing complex (RISC) to miRNA-response elements (MREs) localized on mRNA, which the function of protein production was inhibited. In addition to mRNA, MREs can also be found on nonprotein-coding transcripts, such as circRNA and lncRNA. Each miRNA has many RNA targets, and numerous RNA molecules carry several MREs. This target multiplicity led to the hypothesis that different RNAs compete for limiting miRNAs, thus, acting as ceRNA network. The circRNAs and lncRNAs in the network act as sponges harbor, providing one or more complementary binding sites to miRNAs, which inhibit miRNA binding to mRNAs, thereby improving protein expression and achieving regulatory effect. Especially, circRNAs formed through head-to-tail splicing of exons within the same transcript. Such transcripts were very stable and bound to become a class of powerful ceRNA, playing an important role in normal physiology and disease [33]. The ceRNA plays an important role in the process of CRC including epithelial to mesenchymal transition (EMT), inflammation formation, and so on. In that case, this network proposes the possible reference and direction for cancer therapy and diagnosis biomarkers. However, to further study the ceRNA network in CRC process, more researches should be done.

Our study combines FRG signature with clinical data to construct a prognostic model of CRC. Moreover, we found the relationship between ferroptosis and immunotherapy sensitivity, as well as the potential ceRNA mechanism of the key FRGs in this model. However, there are still some limitations that should be addressed. First, the prognostic model was developed and validated using public databases. Prospective real-world data are needed to validate the clinical efficacy of this model. In addition, the association between ferroptosis-relate genes, immuno-activity, and ceRNA network warrants further investigation.

## 5. Conclusions

In summary, we constructed a novel FRG risk score prognostic model with high accuracy based on 8 FRGs in CRC patients. Our study showed that FRGs can be the potential biomarker for immunotherapy response of CRC. More investigations on the mechanisms of tumor immunity, FRGs, and ceRNA network in CRC are needed to further study.

## Data Availability

The data and materials used to support the findings of this study are available from the corresponding author upon request.
